# The effect of high [K^+^]_o_ on spontaneous Ca^2+^ waves in freshly isolated interstitial cells of Cajal from the rabbit urethra

**DOI:** 10.1002/phy2.203

**Published:** 2014-01-21

**Authors:** Bernard T. Drumm, Gerard P. Sergeant, Mark A. Hollywood, Keith T. Thornbury, Toshio T. Matsuda, Akemichi Baba, Brian J. Harvey, Noel G. McHale

**Affiliations:** 1 Smooth Muscle Research Centre, Dundalk Institute of Technology, DundalkCo. Louth, Ireland; 2 Department of Molecular Medicine, Royal College of Surgeons in Ireland, Beaumont Hospital, Dublin 9, Ireland; 3 Graduate School of Pharmaceutical Sciences, Osaka University, Osaka, Japan

**Keywords:** Calcium oscillations, interstitial cell of Cajal, smooth muscle, sodium/calcium exchange

## Abstract

Interstitial cells of Cajal (ICC) act as putative pacemaker cells in the rabbit urethra. Pacemaker activity in ICC results from spontaneous global Ca^2+^ waves that can be increased in frequency by raising external [K^+^]. The purpose of this study was to elucidate the mechanism of this response. Intracellular [Ca^2+^] was measured in fluo‐4‐loaded smooth muscle cells (SMCs) and ICC using a Nipkow spinning disk confocal microscope. Increasing [K^+^]_o_ to 60 mmol/L caused an increase in [Ca^2+^]_i_ accompanied by contraction in SMCs. Raising [K^+^]_o_ did not cause contraction in ICC, but the frequency of firing of spontaneous calcium waves increased. Reducing [Ca^2+^]_o_ to 0 mmol/L abolished the response in both cell types. Nifedipine of 1 *μ*mol/L blocked the response of SMC to high [K^+^]_o_, but did not affect the increase in firing in ICC. This latter effect was blocked by 30 *μ*mol/L NiCl_2_ but not by the T‐type Ca^2+^ channel blocker mibefradil (300 nmol/L). However, inhibition of Ca^2+^ influx via reverse‐mode sodium/calcium exchange (NCX) using either 1 *μ*mol/L SEA0400 or 5 *μ*mol/L KB‐R7943 did block the effect of high [K^+^]_o_ on ICC. These data suggest that high K^+^ solution increases the frequency of calcium waves in ICC by increasing Ca^2+^ influx through reverse‐mode NCX.

## Abstract (precis)

Pacemaker activity in ICC results from spontaneous global Ca^2+^ waves that can be increased in frequency by raising external [K^+^]. The experiments described support the hypothesis that high K^+^ solution increases the frequency of calcium waves in ICC by increasing Ca^2+^ influx through reverse‐mode NCX.

## Introduction

Spontaneous myogenic tone generated by the smooth muscle wall of the urethra is associated with the occurrence of spontaneous electrical slow waves (Hashitani et al. 1996; Hashitani and Edwards 1999). These events are similar to those which have been recorded in the gastrointestinal tract (GIT) where they originate in interstitial cells of Cajal (ICC) (Sanders 1996; Sanders et al 2006). This led Sergeant et al. (2000) to suggest that a similar pacemaking mechanism might exist in the urethra.

The rabbit urethra contains a subpopulation of cells which are distinct both functionally and morphologically from the bulk smooth muscle cells (SMCs) (Sergeant et al. 2000). These cells (which we will refer to as ICC, by analogy with the cells found in the gastrointestinal tract) are noncontractile, but show regular spontaneous transient depolarizations (STDs) under current clamp conditions. The currents or STICs (spontaneous transient inward currents), which underlie the STDs, are believed to result from activation of Ca^2+^‐dependent Cl^−^ channels by Ca^2+^ released from intracellular stores (Sergeant et al. 2001a,b.

Among the many differences between urethral ICC and SMCs are their responses to raised extracellular potassium. In the former cells there is no evidence of length change (as the cells are noncontractile), but firing frequency of spontaneous calcium waves is greatly increased. In contrast, urethral SMCs respond with a raised intracellular calcium and cell contraction.

The purpose of this study was to investigate the underlying mechanisms of these differing responses.

## Methods

### Cell isolation

All procedures were carried out in accordance with current EU legislation and with the approval of Dundalk Institute of Technology Animal Use and Care Committee. Male and female New Zealand white rabbits (16–20 weeks old) were humanely killed with a lethal injection of pentobarbitone (i.v.). The midurethra was removed and placed in Krebs solution and individual ICC were isolated enzymatically as previously described (Bradley et al. 2006).

### Calcium imaging

Cells were incubated in 2 *μ*mol/L fluo‐4/AM (Molecular Probes, Paisley, UK) in Hanks solution for 30 min at 37°C. They were then washed in Hanks solution and allowed to settle in glass‐bottomed Petri dishes until they had stuck down. Cells were imaged using an iXon 887 EMCCD camera (Andor Technology, Belfast; 512 × 512 pixels, pixel size 16 × 16 *μ*m) coupled to a Nipkow spinning disk confocal head (CSU22, Yokogawa, Japan). A krypton–argon laser (Melles Griot, Zevenar, the Netherlands) at 488 nm was used to excite the fluo‐4, and the emitted light was detected at wavelengths >510 nm. Images were usually acquired at 5 frames/sec. Analysis of recordings was performed using iQ software (Andor, Belfast, UK). Movie files recorded in iQ were converted into a stack of TIFF (tagged image file format) images and imported into Image J (version1.40, National Institutes of Health, MD; http://rsbweb.nih.gov/ij) for post hoc analysis. Prior to analysis, background fluorescence was subtracted from the stack. A single pixel line was drawn along the midaxis of the cell and using the “reslice” function in Image J (NIH, Bethesda, MD) a pseudo–line‐scan image was produced with distance along the cell (*μ*m) on the vertical axis and time (sec) on the horizontal axis. Basal fluorescence was obtained from areas of the cell displaying the most uniform and least intense fluorescence (F_0_). To analyze Ca^2+^ waves, a plot profile was generated by drawing a rectangle over an entire line scan and plotting an intensity profile in Image J. The amplitude of waves was obtained from this plot profile by calculating the difference between basal Ca^2+^ fluorescence and the peak Ca^2+^ fluorescence with amplitude values expressed as ΔF/F_0_. Waves were defined as events which were >25% of the maximum amplitude event during a control period.

Data were expressed as mean ± standard error of the mean (SEM) for the number of experiments (cells) analyzed. In each case, cells were taken from a minimum of two animals. Statistical analysis was performed using either the Student's paired *t*‐test or analysis of variance (ANOVA) with a Tukey multiple comparison test where multiple comparisons were required. In all statistical analyses, *P* < 0.05 was taken as significant.

### Solutions

The solutions used were of the following composition (mmol/L):

*Ca*^*2+*^*‐free Hanks solution (cell isolation):* NaCl (125.0), KCl (5.4), Glucose (10.0), Sucrose (2.9), NaHCO_3_ (4.2), KH_2_PO_4_ (0.4), NaH_2_PO4 (0.3), HEPES (10.0), pH to 7.4 using NaOH. *100 μmol/L Ca*^*2+*^ *Hanks solution*: CaCl_2_.‐2H_2_O (0.1), NaCl (125.0), KCl (5.4), Glucose (10.0), Sucrose (2.9), NaHCO_3_ (4.2), KH_2_PO_4_ (0.4), NaH_2_PO_4_ (0.3), HEPES (10.0), pH to 7.4 using NaOH.

*Normal Hanks solution:* NaCl (125.0), KCl (5.4), Glucose (10.0), Sucrose (2.9), NaHCO_3_ (4.2), KH_2_PO_4_ (0.4), NaH_2_PO_4_ (0.3), MgCl_2_.6H_2_O (0.5), CaCl_2_.2H_2_O (1.8), MgSO_4_ (0.4), HEPES (10.0), pH to 7.4 using NaOH.

*Ca*^*2+*^*‐free Hanks solution (superfusion):* NaCl (125.0), KCl (5.4), Glucose (10.0), Sucrose (2.9), NaHCO_3_ (4.2), KH_2_PO_4_ (0.4), NaH_2_PO_4_ (0.3), MgCl_2_.6H_2_O (2.3), EGTA (5.0), MgSO_4_ (0.4), HEPES (10.0), pH to 7.4 using NaOH. *K*^*+*^ *Hanks solution of 60 mmol/L:* NaCl (70.8), KCl (59.65), Glucose (10.0), Sucrose (2.9), NaHCO_3_ (4.2), KH_2_PO_4_ (0.4), NaH_2_PO_4_ (0.3), MgCl_2_.6H_2_O (0.5), CaCl_2_.2H_2_O (1.8), MgSO_4_ (0.4), HEPES (10.0). pH to 7.4 using NaOH. *Na*^*+*^ *Hanks solution of 75 mmol/L:* NMDG (54.2) NaCl (70.8), KCl (5.4), Glucose (10.0), Sucrose (2.9), NaHCO_3_ (4.2), KH_2_PO_4_ (0.4), NaH_2_PO_4_ (0.3), MgCl_2_.6H_2_O (0.5), CaCl_2_.2H_2_O (1.8), MgSO_4_ (0.4), HEPES (10.0), pH to 7.4 using HCl.

### Drugs

Drugs were made up in dimethyl sulphoxide (DMSO), ethanol, or water depending on solubility. Stock solutions were added to the drug delivery reservoirs containing Hanks solution to make up the final concentrations. Drugs used were as follows: KB‐R7943, Tocris (Bristol, UK); Mibefradil, Sigma (Wicklow, Ireland); Nifedipine, Bayer (Leverkusen, Germany); SEA0400 was synthesized by Taisyo Pharmaceutical Co., Ltd., Saitama, Japan. Mibefradil was water soluble, while SEA0400 and KB‐R7943 were dissolved in DMSO and diluted with Hanks solution to give a final DMSO concentration of 0.1% and 0.05%, respectively. Nifedipine was first dissolved in ethanol and diluted with Hanks solution to give a final ethanol concentration of 0.1%. Control experiments showed that these concentrations of vehicle had no significant effects on the responses measured in this study.

The cell under study was continuously superfused with Hanks solution by means of a close delivery system consisting of a pipette (tip diameter 200 *μ*m) placed approximately 300 *μ*m away. This could be switched, with a dead space time of around 5 sec, to a solution containing a drug. All experiments were carried out at 35–37°C.

## Results

Single ICC isolated from the rabbit urethra displayed regularly occurring Ca^2+^ waves as previously described (Johnston et al. 2005; Sergeant et al. 2006a,b. The mean frequency, amplitude, and spread of these waves, measured under control conditions, in 34 cells were 5.3 ± 0.7 min^−1^, 1.9 ± 0.2 ΔF/F_0_, and 75.8 ± 4.4 *μ*m, respectively.

### The effect of high [K^+^]_o_

**Figure 1.**
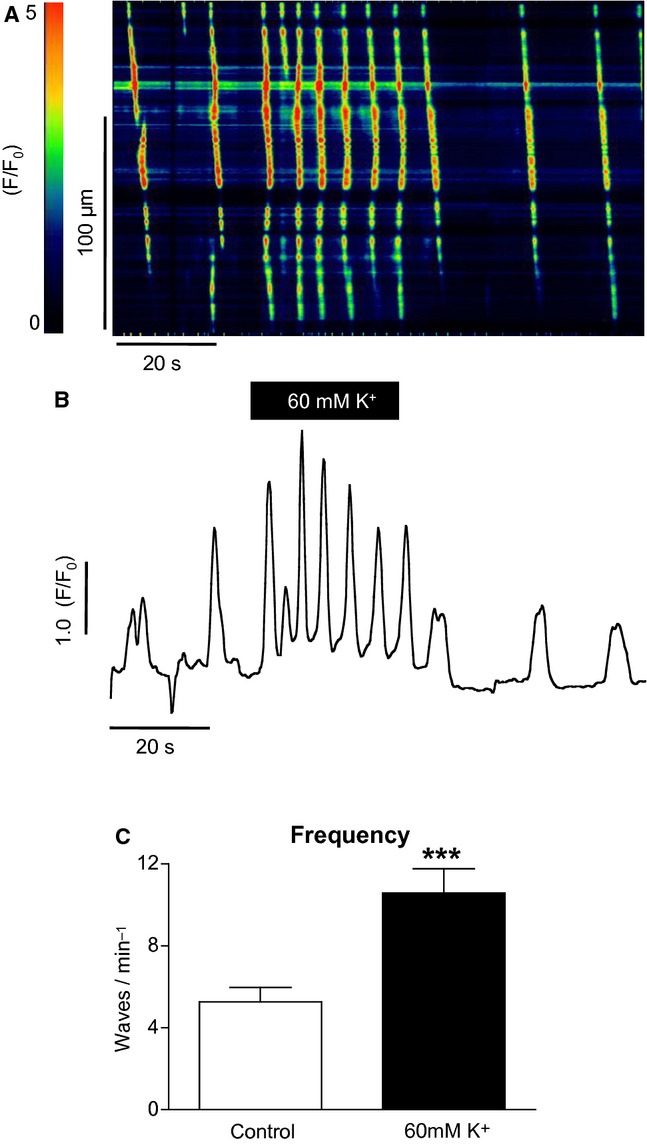
The effect of high [K^+^]_o_ on spontaneous Ca^2+^ waves in a urethral ICC. (A) Representative pseudo–line scan and (B) intensity profile. Oscillation frequency increased from 66 min^−1^ in control conditions to 12 min^−1^ in high [K^+^]_o_. Similar results were observed in a total of 34 cells and these results are summarized in (C).

Figure 1A shows a representative pseudo–line scan showing the effect of high [K^+^]_o_ (60 mmol/L) on spontaneous Ca^2+^ waves in a urethral ICC. In control conditions (5.8 mmol/L [K^+^]_o_), the cell fired spontaneous Ca^2+^ waves at a frequency of ~6 min^−1^. When [K^+^]_o_ was raised to 60 mmol/L the frequency increased to 12 min^−1^. Similar results were observed in a total of 34 cells and this is summarized in Figure 1C. The mean frequency of waves was 5.3 ± 0.7 min^−1^ under control conditions compared to 10.6 ± 1.2 min^−1^ in 60 mmol/L [K^+^]_o_ (*P* < 0.001, paired *t‐*test).

**Figure 2.**
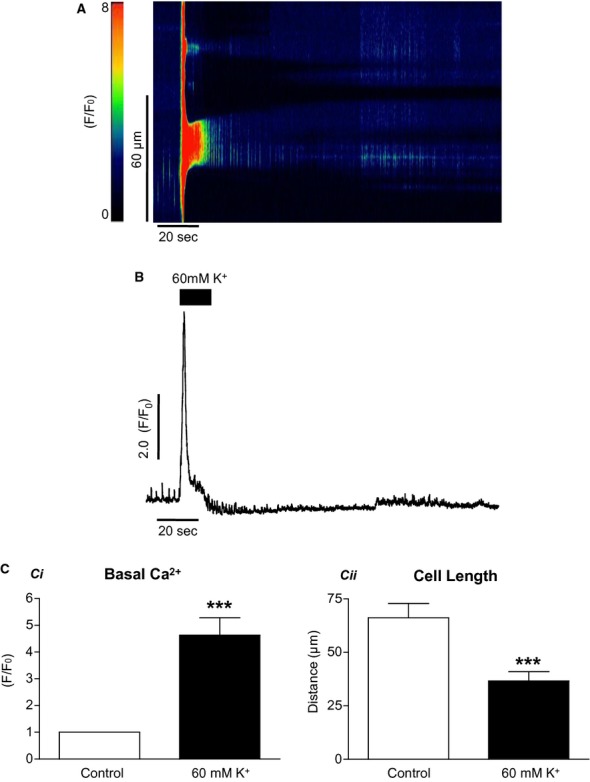
The effect of high [K^+^]_o_ on isolated smooth muscle cells. High [K^+^]_o_ caused a transient rise in [Ca^2+^]_i_ (A and B) and caused the cell to contract. The results of 12 such experiments are summarized in (C). Basal F/F_0_ was increased from 1.0 in control to 4.6 ± 0.7 in high [K^+^]_o_, whereas mean cell length was reduced from 66.2 ± 6.8 to 36.7 ± 4.4 *μ*m. When [K^+^]_o_ was restored to control concentrations, cells relaxed but not quite to control levels.

When this experiment was repeated using SMCs from the rabbit urethra, as seen in Figure 2A, high [K^+^]_o_ caused a transient rise in [Ca^2+^]_i_. In a total of 12 cells, the basal F/F_0_ was increased from 1 ± 0 in control to 4.6 ± 0.7 in high [K^+^]_o_ (*P* < 0.001, paired *t*‐test). Application of high [K^+^]_o_ also caused the SMCs to contract. Mean cell length was reduced from 66.2 ± 6.8 to 36.7 ± 4.4 *μ*m (*P* < 0.001, paired *t*‐test). When [K^+^]_o_ was restored to control concentrations, cells relaxed but not quite to control levels.

### The effect of blocking Ca^2+^ influx

**Figure 3.**
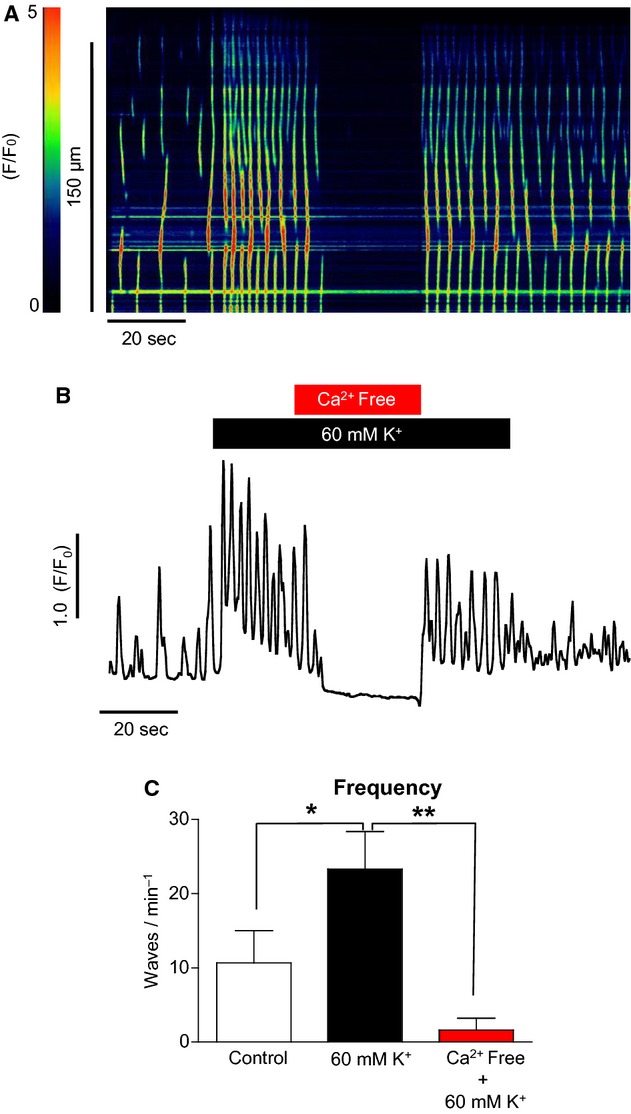
The effect of Ca^2+^‐free Hanks solution on high [K^+^]_o_‐induced Ca^2+^ oscillations in ICC. (A, B) The increase in frequency in response to raised [K^+^]_o_ depends on external Ca^2+^. (C) Summary of four such experiments.

To determine if the effect of high [K^+^]_o_ on Ca^2+^ wave frequency in ICC was due to an effect on Ca^2+^ influx or a direct effect on store release, Ca^2+^‐free Hanks solution was used to prevent Ca^2+^ influx across the plasma membrane. Figure 3A displays a pseudo–line scan from such an experiment. When external [K^+^]_o_ was raised from 5.8 to 60 mmol/L, the frequency of Ca^2+^ waves was increased and this effect was abolished when external Ca^2+^ was removed from the bathing solution. The effects of Ca^2+^‐free Hanks on the high [K^+^]_o_ effect are summarized in Figure 3C. The frequency of waves was increased from 10.7 ± 4.4 min^−1^ in control to 23.3 ± 5 min^−1^ in high [K^+^]_o_ (*P* < 0.05, ANOVA, *n* = 4), and this was reduced to 1.6 ± 1.6 min^−1^ when Ca^2+^ was removed from the external solution (*P* < 0.01, ANOVA, *n* = 4), suggesting that the response was dependent on Ca^2+^ influx.

### The effect of low [Na^+^]_o_

**Figure 4.**
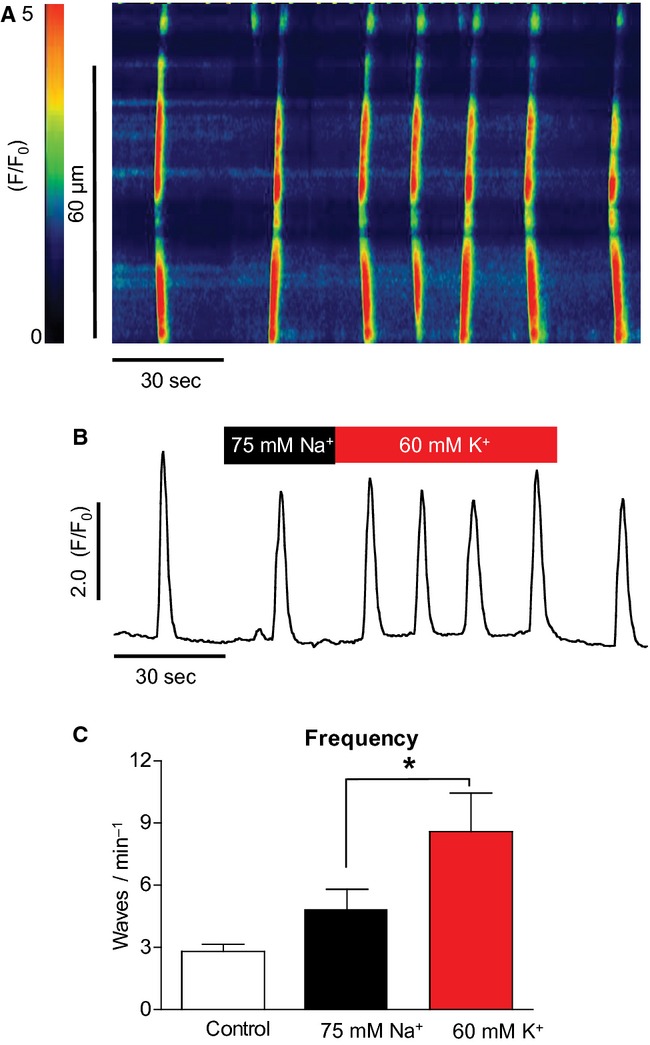
The effect of 75 mmol/L [Na^+^]_o_ on oscillation frequency (A & B). There was a small increase in oscillation frequency on reduction in [Na^+^]_o_, but this was not significant; whereas the subsequent addition of 60 mmol/L [K^+^]_o_ did result in a significant frequency increase (C) (*n* = 8, *P* < 0.05).

In order to prepare the high [K^+^]_o_ solution for the above experiments, NaCl was removed from normal Hanks solution and replaced with KCl. Thus, the [Na^+^]_o_ was decreased from 130 to 75 mmol/L. However, it is known that decreasing [Na^+^]_o_ from 130 to 13 mmol/L increases the frequency of spontaneous Ca^2+^ waves in ICC (Bradley et al. 2006). To ensure that the increase in Ca^2+^ wave frequency was due to the high [K^+^]_o_ and not low [Na^+^]_o_, control experiments were performed in which the [K^+^]_o_ was maintained at control levels (5.8 mmol/L) and the [Na^+^]_o_ was decreased to 75 mmol/L and replaced with equimolar *N*‐methyl d‐glucamine (NMDG). A typical experiment using this protocol in ICC is shown in Figure 4A. Summarized data for eight such experiments are shown in Figure 4C. When [Na^+^]_o_ was lowered in this way, the frequency of Ca^2+^ waves increased from 2.8 ± 0.3 in control to 4.7 ± 1.1 min^−1^ in 75 mmol/L Na^+^, but this was not significant (ANOVA). Subsequent addition of 60 mmol/L K^+^ solution significantly increased the frequency of Ca^2+^ waves to 8.6 ± 1.9 min^−1^ (*P* < 0.05, ANOVA). Thus, it seemed that lowering [Na^+^]_o_ to 75 mmol/L alone was insufficient to significantly increase wave frequency.

**Figure 5.**
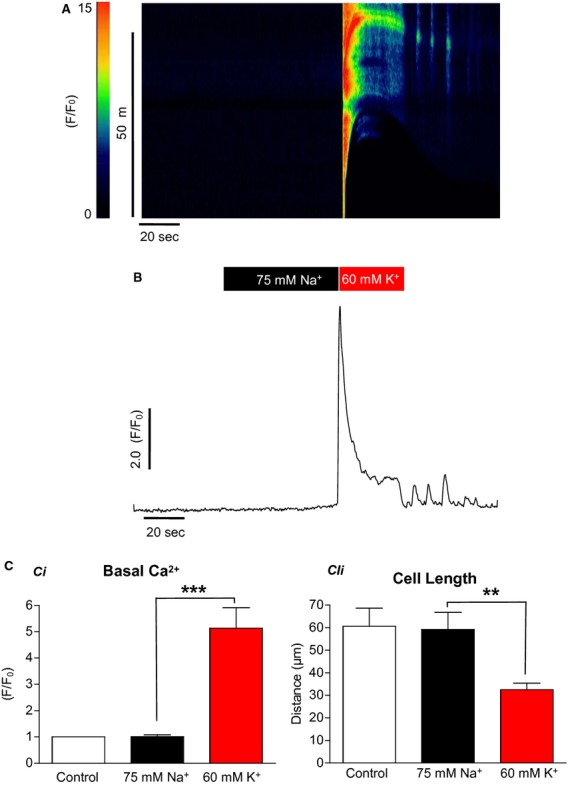
The effect of 75 mmol/L [Na^+^]_o_ on urethral smooth muscle cells (A & B). Reduction in [Na^+^]_o_ to 75 mmol/L did not increase the calcium signal nor did it cause the cells to contract. This was in contrast to the effects of increasing [K^+^]_o_ to 60 mmol/L (C) (*n* = 6, **P < 0.01, ***P < 0.001).

The above protocol was then repeated on SMCs; a representative experiment is shown in Figure 5A. Decreasing [Na^+^]_o_ from 130 to 75 mmol/L with Na^+^ replaced with NMDG did not cause a significant rise in [Ca^2+^]_i_. Summary data in Figure 5C show that in a total of six cells, the basal F/F_0_ under 75 mmol/L Na^+^ conditions was not significantly affected (1 to 1 ± 0.1, ANOVA). When [K^+^]_o_ was then increased to 60 mmol/L, the F/F_0_ increased to 5.1 ± 0.8 (*P* < 0.001, ANOVA). Similarly, decreasing [Na^+^]_o_ to 75 mmol/L did not affect the cell length; however, when [K^+^]_o_ was raised to 60 mmol/L the cell contracted from 59.2 ± 7.5 to 32.5 ± 2.9 *μ*m (*P* < 0.01, ANOVA).

### Role of L‐type Ca^2+^ channels

**Figure 6.**
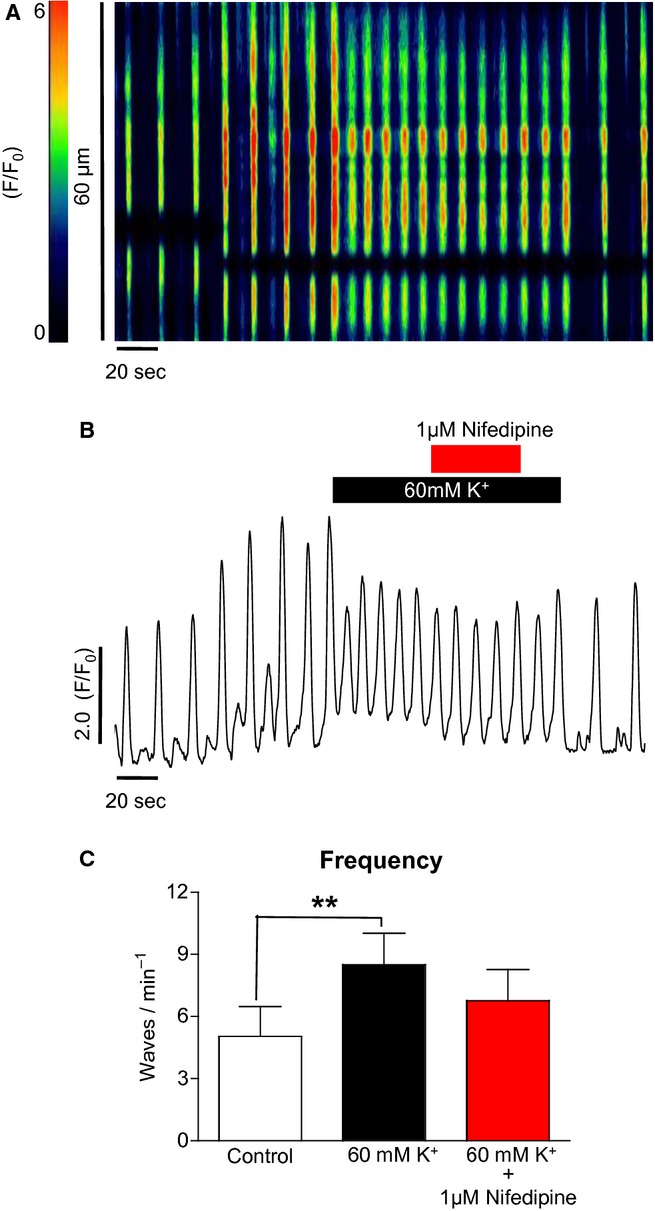
The effect of nifedipine on high [K^+^]_o_‐induced Ca^2+^ oscillations in ICC (A & B). Nifedipine (1 *μ*mol/L) caused only a small reduction in frequency in the presence of high [K^+^]_o_. In five such experiments, the reduction was not significant (C).

Application of high [K^+^]_o_ would presumably depolarize ICC and could activate voltage‐gated Ca^2+^ channels. Their activation could enhance influx and consequently increase wave frequency. To test this, we examined the effect of nifedipine on the response. When 1 *μ*mol/L nifedipine was applied, the increased wave frequency in response to high K^+^ was not significantly reduced. Summarized data for five such experiments in Figure 6C show that the frequency of high K^+^‐induced waves was not significantly reduced (8.5 ± 1.5 to 6.8 ± 1.5 min^−1^, ns, ANOVA).

**Figure 7.**
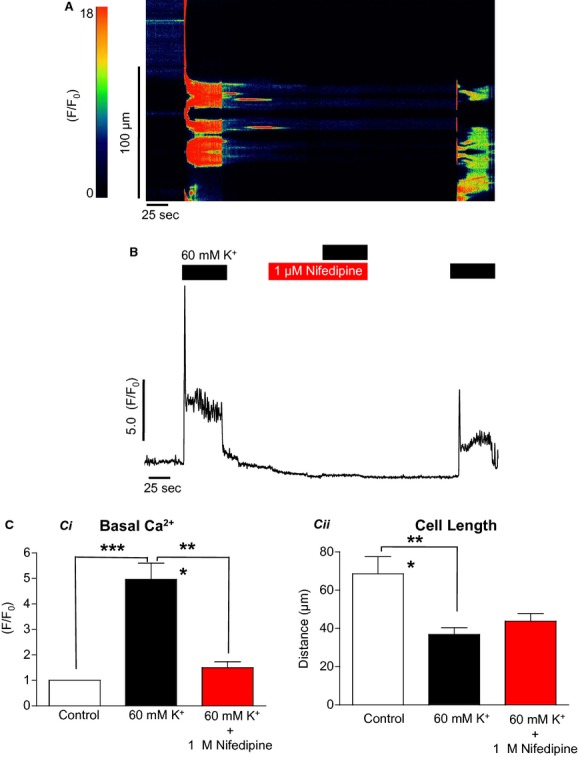
The effect of nifedipine on high [K^+^]_o_‐induced contraction in isolated smooth muscle cells (A & B). In contrast to the effects seen in ICC, nifedipine was very effective in blocking the increase in [Ca^2+^]_i_ and the contraction elicited by high [K^+^]_o_ (C) (*n* = 8, ***P < 0.001).

The above results from ICC were in contrast to those found in urethral SMCs. Figure 7A shows a typical experiment with a SMC in which high [K^+^]_o_ caused a rise in [Ca^2+^]_i_ and this was substantially attenuated by 1 *μ*mol/L nifedipine. Summarized data in Figure 7C show that in eight SMCs the F/F_0_ increase induced by high K^+^ was reduced from 5 ± 0.6 to 1.5 ± 0.2 F/F_0_ by nifedipine (*P* < 0.001, ANOVA).

### Role of T‐type Ca^2+^ channels

**Figure 8.**
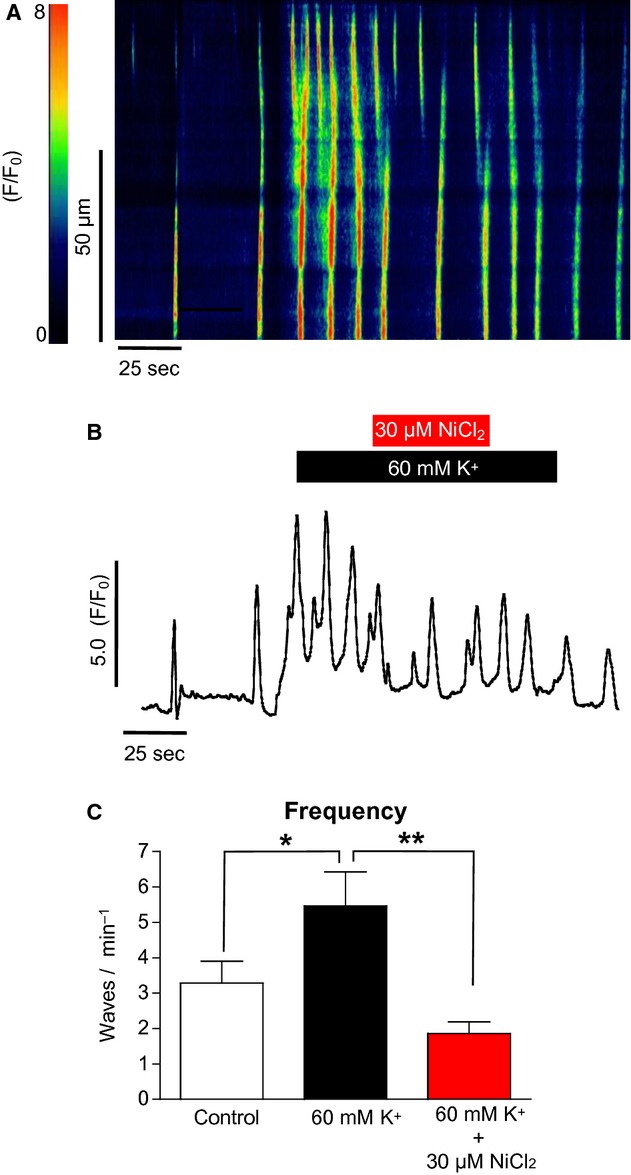
The effect of NiCl_2_ on high [K^+^]_o_‐induced Ca^2+^ oscillations in ICC (A & B). The increase in oscillation frequency in response to high [K^+^]_o_ was significantly depressed by NiCl_2_, suggesting a possible role for influx via T‐type channels. (C) Summary of four such experiments (*P < 0.05, **P < 0.01).

The above results indicated that L‐type channels did not play a major role in mediating the high K^+^ response in ICC. However, another possible mechanism was Ca^2+^ influx via the voltage‐dependent T‐type Ca^2+^ channel. When 30 *μ*mol/L NiCl_2_ (a known blocker of T‐type channels) was applied to ICC as shown in Figure 8A, the high K^+^ effect was inhibited. Summary data in Figure 8C show that 30 *μ*mol/L NiCl_2_ significantly reduced the frequency of waves in high [K^+^]_o_ conditions from 5.5 ± 1 to 1.9 ± 0.3 min^−1^ (*P* < 0.01, ANOVA, *n* = 4).

**Figure 9.**
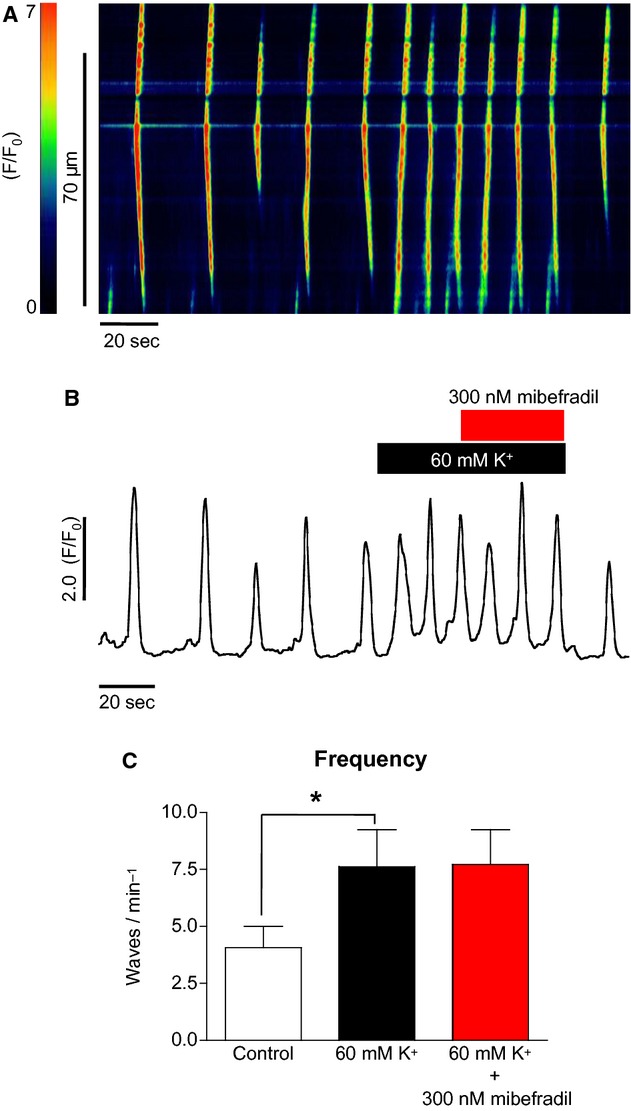
The effect of mibefradil on high [K^+^]_o_‐induced Ca^2+^ oscillations in ICC (A & B). Application of the specific T‐type channel blocker mibefradil (A) did not significantly depress the high K^+^ response in ICC (C) (*n* = 4).

However, when a more specific T‐type channel blocker mibefradil (300 nmol/L) was applied (Fig. 9A), the high K^+^ response in ICC was not significantly reduced. This is summarized in Figure 9B; the frequency of Ca^2+^ waves under high [K^+^]_o_ was little changed (7.6 ± 1.6 to 7.7 ± 1.5 min^−1^ in mibefradil, ns, ANOVA, *n* = 4).

### Role of reverse‐mode sodium/calcium exchange

Ni^2++^ significantly reduced the frequency of waves in high [K^+^]_o_ conditions and the results in Figure 9 suggested that the inhibitory effect of NiCl_2_ was due to a mechanism other than that of blocking T‐type channels. NiCl_2_ is also known to block sodium/calcium exchange (NCX) (Blaustein and Lederer 1999). As reverse‐mode NCX has been indicated to be an important Ca^2+^ influx pathway in urethral ICC (Bradley et al. 2006), it was decided to investigate this pathway as a mediator of the high K^+^ response.

**Figure 10.**
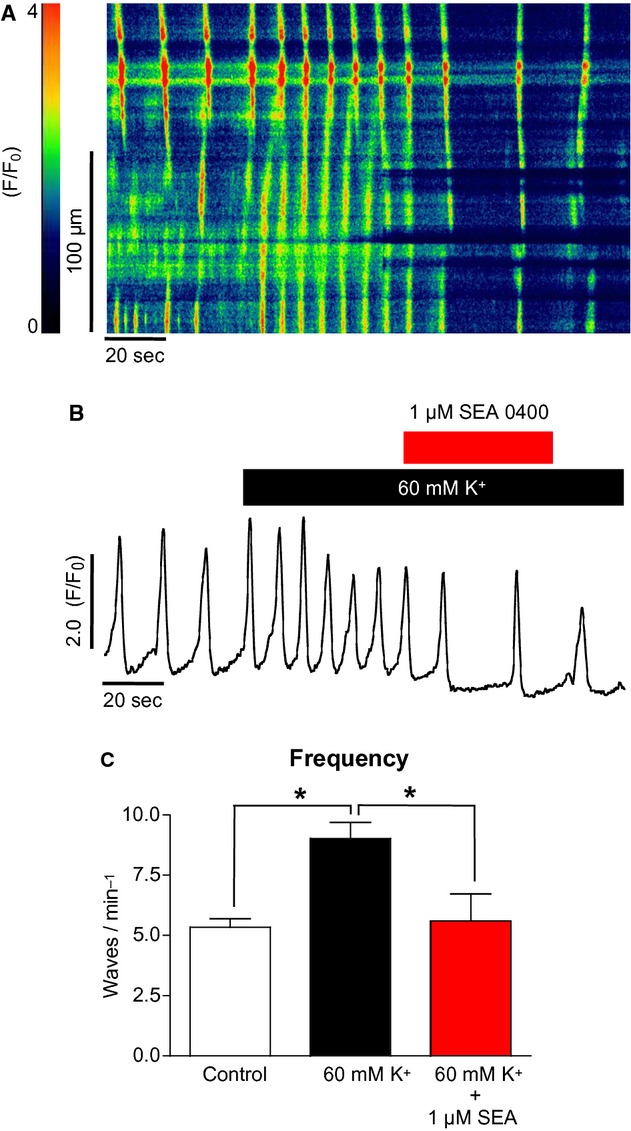
The effect of SEA0400 on high [K^+^]_o_‐induced Ca^2+^ oscillations in ICC (A & B). The increase in oscillation frequency in response to high [K^+^]_o_ was significantly depressed by the reverse‐mode NCX blocker SEA0400. (C) Summary of six such experiments (*P < 0.05).

Figure 10A shows the effect of high [K^+^]_o_ before and after addition of the NCX reverse‐mode blocker SEA0400 (Hotta et al. 2007) (1 *μ*mol/L). Addition of the latter drug completely reversed the high [K^+^]_o_‐induced frequency increase. This is summarized in Figure 10C; Ca^2+^ wave frequency under high [K^+^]_o_ conditions was reduced from 9 ± 0.7 to 5.6 ± 1.1 min^−1^ by SEA0400 (*P* < 0.05, ANOVA, *n* = 6).

**Figure 11.**
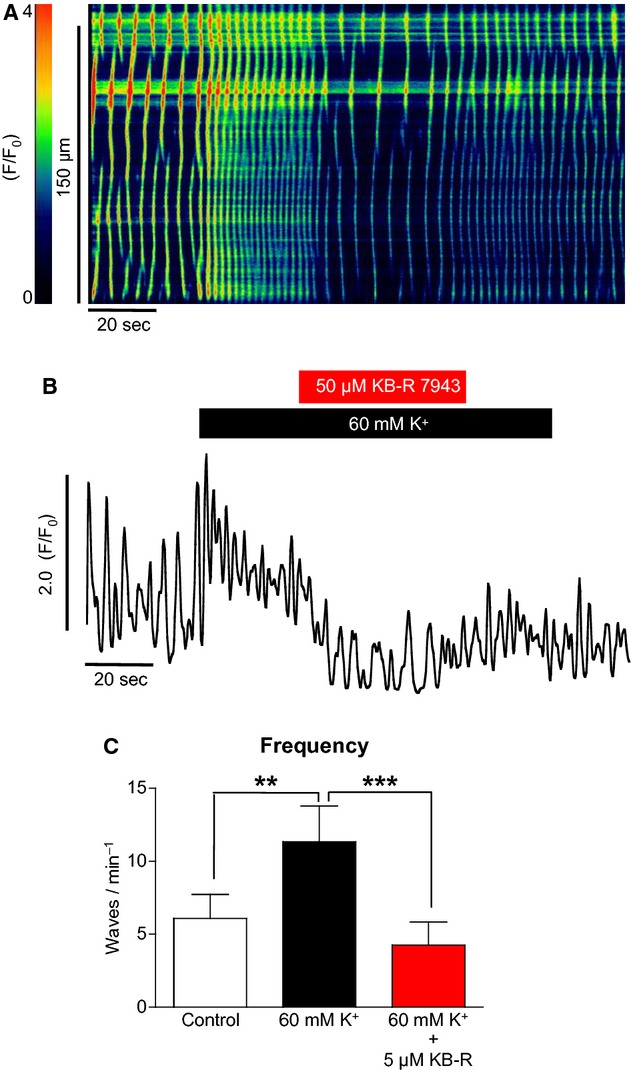
The effect of KB‐R7943 on high [K^+^]_o_‐induced Ca^2+^ oscillations in ICC (A & B). The increase in oscillation frequency in response to high [K^+^]_o_ was also significantly depressed by KB‐R7943, suggesting a role for reverse‐mode NCX as a calcium influx pathway. (C) Summary of six such experiments (**P < 0.01, ***P < 0.001).

Figure 11A shows that the specific reverse‐mode NCX inhibitor KB‐R7943 had a similar effect on the high [K^+^]_o_ response in ICC. The increase in Ca^2+^ wave frequency was completely reversed by KB‐R7943. Summary data in Figure 11C show that KB‐R reduced the high K^+^‐induced frequency of waves from 11.3 ± 2.5 to 4.3 ± 1.6 min^−1^ (*P* < 0.001, ANOVA, *n* = 6).

## Discussion

The results from this study demonstrate that the frequency of spontaneous Ca^2+^ oscillations in rabbit urethral ICC was significantly increased by high [K^+^]_o_ solutions. In agreement with our observations, increasing [K^+^]_o_ to 60 mmol/L in ICC from rabbit portal vein caused a transient increase in [Ca^2+^]_i_ and also contracted SMCs from the same tissue (Povstyan et al. 2003).

The 60 mmol/L K^+^ solution used in this study involved substituting K^+^ for Na^+^. It has been shown in previous studies that lowering [Na^+^]_o_ to 13 mmol/L can itself increase the frequency of Ca^2+^ oscillations in urethral ICC (Bradley et al. 2006). However, when ICC were bathed in a 75 mmol/L [Na^+^]_o_ solution only a slight increase in wave frequency was observed and this was not significant. Similarly, in SMCs 75 mmol/L [Na^+^]_o_ did not induce a rise in Ca^2+^ or cause contraction of the cell. Lowering the [Na^+^]_o_ to 75 mmol/L (with NMDG substitution) will increase the driving force for calcium (as a result of making E_NCX_ more negative), but the present results suggest that this is not sufficient to significantly increase calcium influx either in ICC or SMCs. Replacing the [Na^+^]_o_ with K^+^, on the other hand, has a much greater effect on driving force (affecting both E_NCX_ and membrane potential) with the observed significant effects on calcium influx in both cell types.

Elevation of [K^+^]_o_ appeared to increase the frequency of oscillations by increasing Ca^2+^ influx into the cells. This was supported by the fact that removal of Ca^2+^ from the external bathing solution abolished the high K^+^ effect. These data demonstrated that the high K^+^ effect on Ca^2+^ waves was via increased Ca^2+^ influx and not a direct effect on ER store release. The pathway for Ca^2+^ influx was then investigated pharmacologically.

Ca^2+^ influx through L‐type channels was a major contributor to the high [K^+^]_o_ effect in SMCs but not in ICC. Blocking the channels with nifedipine abolished the high [K^+^]_o_ effect in SMCs but not in ICC. Previous work from our laboratory showed that Ca^2+^ influx in ICC through L‐type Ca^2+^ channels has little affect on Ca^2+^ waves (Johnston et al. 2005) and this was further supported by our study.

When 30 *μ*mol/L Ni^2+^ was used to block T‐channels, the burst of oscillations induced by high [K^+^]_o_ was significantly inhibited and wave frequency reverted to near control levels. These experiments suggested that the T‐type Ca^2+^ channel may play a role in mediating Ca^2+^ influx in ICC. However, when a more specific T‐type channel blocker, mibefradil (Strege et al. 2005), was applied, the frequency of oscillations only slightly decreased suggesting that Ni^2+^ may have acted by inhibiting another pathway.

Ni^2+^ is also known to be a nonselective blocker of NCX (Rebolledo et al. 2005). NCX is normally thought of as a Ca^2+^ extrusion mechanism, however, it is known to act as a bidirectional antiporter (Blaustein and Lederer 1999). Reverse‐mode NCX has been previously demonstrated to be an important Ca^2+^ entry pathway in many cell types including ventricular myocytes (Laflamme and Becker 1996), dog and ferret erythrocytes (Blaustein and Lederer 1999), detrusor SMCs (Wu and Fry 2001), human bone marrow–derived mesenchymal stem cells (Kawano et al. 2003), HEK 293 cells (Rosker et al. 2004), human umbilical artery myocytes (Rebolledo et al. 2005), pulmonary arteriole myocytes (Zhang et al. 2005), and murine portal vein myocytes (Saleh et al. 2005).

Reverse‐mode NCX has also been indicated as a major Ca^2+^ influx pathway in urethral ICC (Bradley et al. 2006) and also ICC from the murine small intestine where whole‐tissue imaging demonstrated that Ca^2+^ oscillations were blocked by KB‐R7943 but not by nicardipine or mibefradil (Lowie et al. 2011). This latter study also used immunolabeling of IP_3_Rs and NCX to show that both proteins were highly expressed in ICC, suggesting that they formed a microdomain and that influx via reverse‐mode NCX regulated ICC Ca^2+^ signaling via modulation of IP_3_Rs. Under our experimental conditions, E_NCX_ has been calculated at −72 mV and membrane potentials positive to this value would result in the exchanger switching to reverse mode and allowing Ca^2+^ influx rather than extrusion. If we assume that intracellular sodium was to oscillate between 10 and 20 mmol/L, then E_NCX_ would vary from −53 to −84 mV (assuming a constant intracellular Ca^2+^ of 1 nmol/L). This means that, if we neglect changes in other ion concentrations, NCX would sometimes be in forward and sometimes in reverse mode. The membrane potential of urethral ICC and SMC has been estimated to be approximately −60 mV (Bradley et al. 2006). In this study we calculated that if ICC and SMC had a resting membrane potential of −60 mV, increasing [K^+^]_o_ from 5.8 to 60 mmol/L would shift this potential from −60 to −22 mV. This shift brought the membrane potential of the cell into the window of L‐type channel activation. The activation of L‐type channels could then lead to increased influx and this mechanism would explain the effects of high [K^+^]_o_ seen in urethral SMCs. However, the high [K^+^]_o_ effect seen in urethral ICC was not due to influx through L‐type channels. Shifting the membrane potential of ICC from −60 to −22 mV would also increase the driving force of reverse‐mode NCX as the membrane potential moves positively away from E_NCX_. When specific blockers of reverse‐mode NCX such as SEA0400 (Matsuda et al. 2001) and KB‐R7943 were applied to ICC, the high [K^+^]_o_ effect was inhibited and oscillation frequency returned to control levels. This supports the view that in urethral ICC the effects of high [K^+^]_o_ were due to increased Ca^2+^ influx via reverse‐mode NCX.

In conclusion, we have shown that urethral ICC have very different properties from those of urethral SMCs. Thus, raising [K^+^]_o_ causes an influx of Ca^2+^ through L‐type calcium channels in SMCs and this is followed by cell contraction. This is in contrast to the response in urethral ICC, where high [K^+^]_o_ increases the frequency of Ca^2+^ oscillations by increasing Ca^2+^ influx across the plasma membrane. Our results suggest that the main influx pathway for this latter effect is reverse‐mode NCX.

## Acknowledgments

The studies using SEA0400 were done in collaboration with Professor Matsuda and Doctor Baba of Osaka University.

## Conflict of Interest

None declared.
